# Harlequin Syndrome in Venoarterial ECMO and ECPELLA: When ECMO and Native or Impella Circulations Collide — A Comprehensive Review

**DOI:** 10.31083/RCM39992

**Published:** 2025-08-26

**Authors:** Debora Emanuela Torre, Carmelo Pirri

**Affiliations:** ^1^Department of Cardiac Anesthesia and Intensive Care Unit, Cardiac Surgery, Ospedale dell’Angelo, 30174 Venice, Italy; ^2^Department of Neurosciences, Institute of Human Anatomy, University of Padova, 35121 Padova, Italy

**Keywords:** Harlequin syndrome, differential hypoxia, North-South syndrome, veno-arterial extracorporeal membrane oxygenation, ECPELLA configuration, cardiogenic shock management, retrograde ECMO flow, aortic watershed phenomenon, Impella device

## Abstract

Harlequin syndrome, also known as differential hypoxia (DH) or North-South syndrome, is a serious complication of femoro-femoral venoarterial extracorporeal membrane oxygenation (V-A ECMO). Moreover, Harlequin syndrome is caused by competing flows between the retrograde oxygenated ECMO output and the anterograde ejection of poorly oxygenated blood from the native heart. In the setting of impaired pulmonary gas exchange, the addition of an Impella device (ECPELLA configuration), although beneficial for ventricular unloading and hemodynamic support, may further exacerbate this competition and precipitate DH. This narrative review synthesizes current evidence on the pathophysiology, diagnostic strategies, and management of DH in patients supported with V-A ECMO or with ECPELLA. Meanwhile, the timely detection of Harlequin syndrome is essential to prevent cerebral and myocardial hypoxia. Current diagnostic approaches include right radial arterial pressure monitoring, multisite arterial blood gas analysis, cerebral oximetry, and echocardiographic evaluation of flow dynamics. Interestingly, emerging tools such as contrast-enhanced ultrasound (CEUS) and suprasternal transthoracic echocardiography (TTE) show promise for non-invasive bedside identification of flow competition. However, further management of DH requires tailored strategies aimed at restoring adequate oxygen delivery while preserving sufficient ventricular ejection or Impella support. Moreover, circuit reconfiguration remains a key rescue option when conventional optimization fails. This review highlights that successful treatment depends on integrating real-time physiological data with a dynamic understanding of circulatory support, emphasizing the need for multidisciplinary expertise in managing this complex syndrome.

## 1. Introduction 

The indication for veno-arterial extracorporeal membrane oxygenation (V-A ECMO) has progressively expanded over the years. Concurrently, as evidenced by the Extracorporeal Life Support Organization (ELSO) registry, there has been a significant increase in the annual implantation of mechanical circulatory support devices. One of the most commonly used cannulation strategies is femoro-femoral V-A ECMO (F-F V-A ECMO), where a venous cannula drains deoxygenated blood from the cavo-atrial junction, while an arterial cannula delivers oxygenated blood into the femoral artery. This retrograde flow perfuses the systemic circulation, providing cardiac support by bypassing both the heart and the lungs [1]. Despite its hemodynamic efficacy, peripheral V-A ECMO can lead to differential hypoxia (DH) in the presence of respiratory failure [2]. This condition, also known as Harlequin syndrome or dual circulation syndrome or North-South syndrome, arises when desaturated blood ejected by the recovering left ventricle enters the ascending aorta and competes with retrograde ECMO flow. The consequence is uneven regional oxygenation, where hypoxemic blood preferentially perfuses the upper body, including the coronary and cerebral circulations, while the lower body is supplied with well-oxygenated blood from the ECMO circuit [3]. Clinical signs may include upper body cyanosis with preserved lower limb perfusion, resembling the characteristic color disparity of DH. If unrecognized, DH can result in cerebral and myocardial ischemia, warranting prompt interventions such as optimization of ventilatory settings, changes in cannulation strategy, or conversion to central ECMO [4]. The Impella device, a percutaneous, catheter-based left ventricular assist device, is often combined with V-A ECMO in the ECMO with Impella support (ECPELLA) configuration, especially in patients with profound myocardial dysfunction [5, 6, 7, 8, 9]. Impella® CP (Abiomed, Danvers, MA, USA) operates as an axial flow pump, typically inserted retrogradely across the aortic valve via the femoral artery, with its inflow positioned in the left ventricular cavity and its outflow in the ascending aorta. By continuously aspirating blood from the left ventricle and expelling it into the systemic circulation, it reduces end diastolic pressure, wall stress and pulmonary capillary wedge pressure, thereby preventing ventricular distension and secondary pulmonary edema, which are common complications of V-A ECMO in the absence of effective left ventricular ejection. This ventricular unloading enhances myocardial recovery by reducing oxygen consumption and alleviating the deleterious effects of sustained left ventricular distension [10, 11, 12, 13].

Despite its hemodynamic benefits, ECPELLA can contribute to DH in patients with severe pulmonary impairment, by enhancing delivery of poorly oxygenated blood to the aortic arch and coronary vessels. In such cases, the brain and the heart may suffer hypoxic stress despite apparently adequate systemic perfusion. Recognition of this risk is essential and may necessitate targeted adjustments in ventilatory, ECMO, and circulatory management.

## 2. Literature Review

**Fig. 1.**
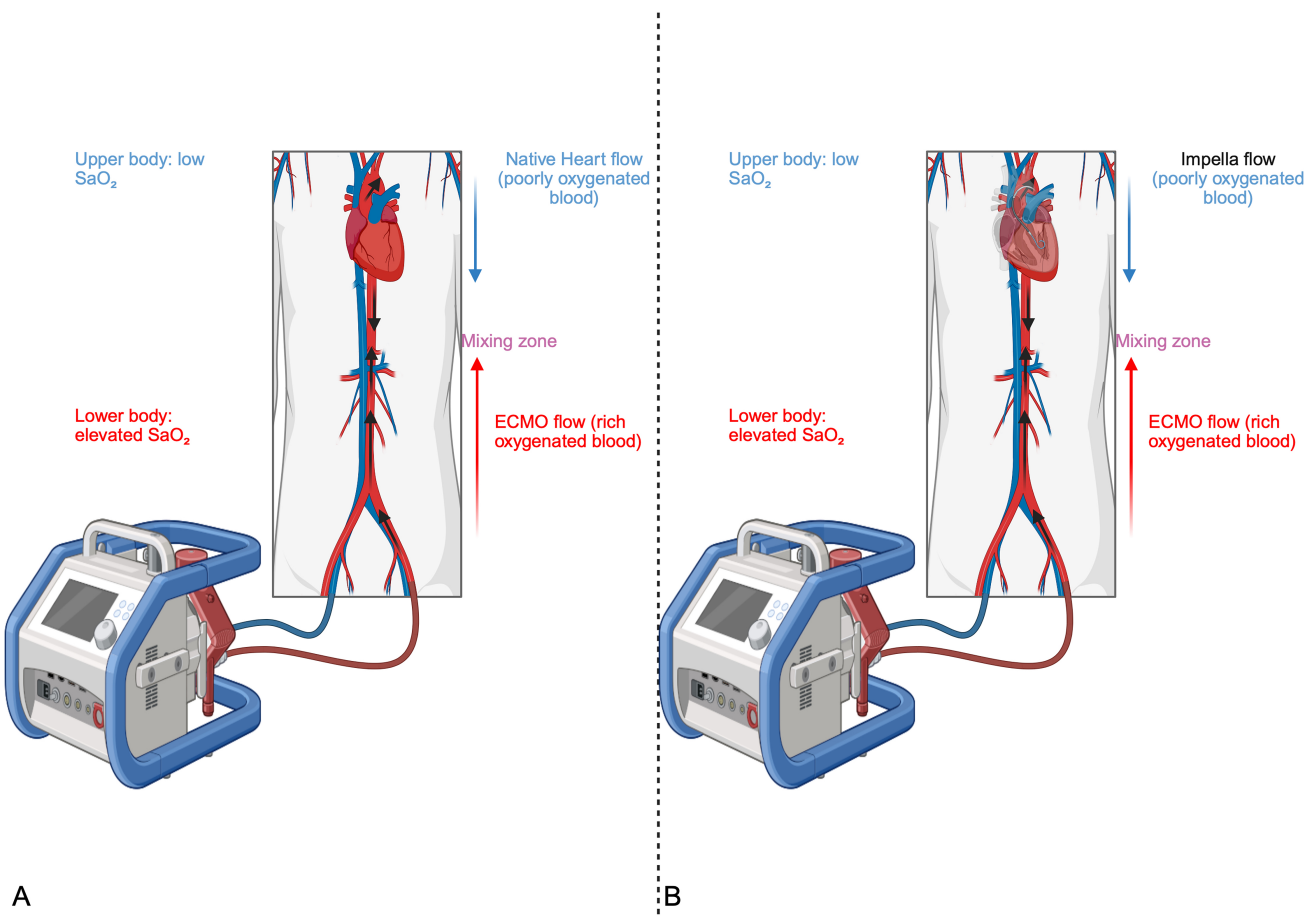
**Differential hypoxia in V-A ECMO (A) and in ECPELLA (B) configurations**. (A) Schematic representation of competitive blood flow dynamics between native cardiac output and retrograde ECMO flow, leading to differential hypoxia (Harlequin/North-South syndrome) in the context of concomitant respiratory failure. Oxygenated blood from the ECMO circuit preferentially perfuses the distal aorta and lower body, while deoxygenated blood from the failing native heart supplies the aortic arch and the upper body, creating a watershed zone of mixed circulation. This phenomenon results in cerebral hypoxemia (due to desaturated native cardiac output) and coronary malperfusion, posing a critical challenge in ECMO management. (B) Schematic representation of antagonistic perfusion patterns during concomitant microaxillary Impella and F-F V-A ECMO support. Deoxygenated blood ejected by the Impella (SaO_2_ <65%, Impella pump flow 1.5 L/min*m^2^) preferentially supplies the aortic arch branches (brachiocephalic, left carotid and subclavian arteries) and thoracic aorta, while oxygenated ECMO retrograde flow (SaO_2_ >95%) supplies abdominal aorta. The mixing zone occurs at the aortic level, where antegrade Impella and retrograde ECMO flows collide, with positional variability dictated by the Impella:ECMO flow ratio (e.g., proximal shift with higher ECMO flows). Risk of upper body and cerebral hypoxia and coronary malperfusion due the Impella-dependent hypoxic flow, contrasted with preserved lower body oxygenation. SaO_2_, oxygen saturation of arterial blood; F-F V-A ECMO, femoro-femoral veno-arterial extracorporeal membrane oxygenation; ECPELLA, ECMO with Impella support. This figure was created with BioRender (http://www.biorender.com/).

While V-A ECMO provides essential hemodynamic and respiratory support, it is associated with several complications, including bleeding, thromboembolic events, vascular injury, infections, and DH [14]. DH is defined as a significant disparity in oxygen saturation (SaO_2_) between the upper and the lower body regions [15]. This phenomenon is particularly evident in femoro-femoral V-A ECMO configurations, where retrograde ECMO perfusion via the iliac artery competes with desaturated blood ejected by the left ventricle (LV). The resulting mixing zone (M-zone or watershed zone) within the aorta creates a dual circulation pattern: the upper body is predominantly perfused by native cardiac output, whereas the lower body receives ECMO-derived oxygenated blood [4] (Fig. 1A). In cases of severe pulmonary dysfunction, the lungs fail to oxygenate venous return adequately, leading to critically low SaO_2_ in the coronary and cerebral circulation despite maintained systemic perfusion. This scenario, termed fulminant differential hypoxia (FDH), can culminate in cerebral and myocardial ischemia [16, 17], underscoring the importance of early recognition and targeted intervention.

### 2.1 Physiopathology of Differential Hypoxia

The ECMO circuit generates hyperoxygenated post-oxygenator blood (S postO_2_ = 100%) with a supra-physiologic dissolved oxygen content. Oxygen delivery (DO_2_) via ECMO is primarily determined by pre-oxygenator saturation (SpreO_2_), hemoglobin concentration (Hb) and pump flow rate (Q ECMO). In regions perfused by ECMO, venous return exhibits elevated saturation. If this highly saturated venous blood recirculates to the right heart, and enters the right ventricle, the pulmonary arterial saturation (SPaO_2_) and subsequent systemic arterial saturation (SaO_2_) increase (the latter due to the higher saturation of the blood ejected from left ventricle). However, inadequate mixing in the right atrium, due to cannula positioning or design, may prevent a concomitant increase in SpaO_2_ and SaO_2_ [4]. Before ECMO initiation, critically ill patients demonstrate uniform SaO_2_ across all vascular beds, with comparable superior and inferior vena cava saturations (SsvcO_2_ = inferior vena cava oxygen saturation (SivcO_2_)) [18]. DH arises when desaturated LV output enters the ascending aorta while oxygenated ECMO flow is infused downstream (e.g., via the femoral artery), creating parallel circulation with discordant SaO_2_ levels dictated by pulmonary and oxygenator function, respectively. The lower body, perfused by ECMO, generates venous return with elevated SivcO_2_. In severe pulmonary failure, the lungs act as a passive conduit between the right and the left heart, rendering upper body SaO_2_ (including coronary arteries and brain) equivalent to mixed venous saturation, or more correctly, similar to SpaO_2_. Consequently, SsvcO_2_ < SivcO_2_ reflects differential oxygen extraction [19, 20, 21].

### 2.2 Clinical Assessment and Diagnosis 

**Fig. 2.**
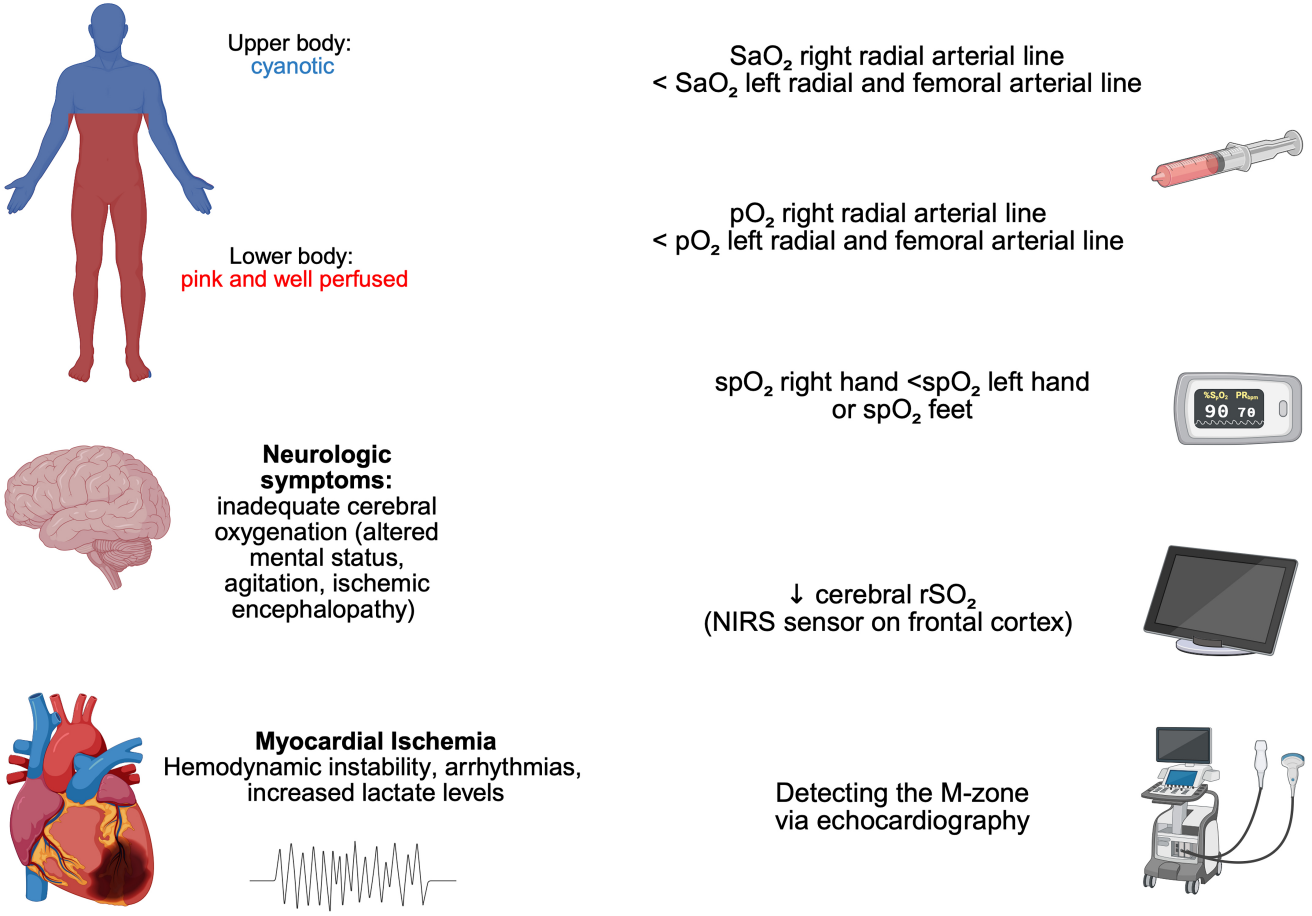
**Clinical assessment and diagnostic criteria for Differential Hypoxia (DH) in Veno-arterial Extracorporeal Membrane Oxygenation (V-A ECMO)**. DH is characterized by competing circulations, with oxygen-depleted native cardiac output perfusing the upper body and ECMO-derived oxygenated flow supplying the lower body. The hallmark clinical sign is a visible contrast between cyanotic upper body tissue and well-perfused lower extremities. Additional findings include neurological deterioration (rSO_2_: <50%, agitation, seizures), and myocardial ischemia (arrhythmias, ST-segment elevation, lactate rise). Diagnosis is supported by oxygenation gradients: right radial arterial blood gases show lower SaO_2_ and pO_2_ compared to left radial and femoral samples; pulse oximetry reveals similar discrepancies between right hand and contralateral upper limb or lower limb values. Echocardiography is pivotal for identifying the mixing zone and monitoring perfusion dynamics. SaO_2_, oxygen saturation of arterial blood; pO_2_, arterial oxygen partial pressure; spO_2_, peripheral oxygen saturation; rSO_2_, regional oxygen saturation; NIRS, near infrared spectroscopy; ↓, decrease. This figure was created with BioRender (http://www.biorender.com/).

Clinically, DH presents with paradoxical central hypoxemia despite preserved lower limb oxygenation. Patients may exhibit cyanosis of the upper extremities, head and neck while the lower body remains well perfused. Neurologic symptoms, such as altered mental status, agitation (if patient is not sedated), or ischemic encephalopathy, may result from inadequate cerebral oxygenation. Myocardial ischemia can develop if coronary perfusion is compromised, leading to hemodynamic instability, arrhythmias, or increased lactate levels [22]. Arterial blood gas analysis typically reveals a discrepancy between sampling sites, with the right radial or brachiocephalic artery demonstrating substantially lower SaO_2_ than both the left radial and femoral arteries. DH can be objectively identified by measuring SaO_2_ at multiple anatomical sites. For surveillance of cerebral hypoxia in DH, continuous pulse oximetry is preferentially applied to the right upper extremity, supplemented by intermittent arterial blood gas sampling from the right radial artery [23]. This monitoring strategy is physiologically justified by the vascular anatomy: the brachiocephalic trunk (innominate artery) represents the first major aortic branch distal to coronary ostia, making right upper extremity saturation measurements the most sensitive clinical indicator of proximal aortic oxygen tension. Consequently, right sided values provide the earliest warning of cerebral hypoperfusion, as the right subclavian and common carotid arteries originate from this most proximal supra-aortic vessel. It should also be noted that non invasive monitoring of regional cerebral oxygen saturation (rSO_2_), obtained via near infrared spectroscopy (NIRS) with sensors positioned on the frontal cortex, may serve as an additional valuable tool in detecting cerebral hypoxia. This modality provides continuous, real-time assessment of cerebral oxygenation, complementing conventional pulse oximetry and arterial blood gas analysis in the evaluation of DH [24] (Fig. 2).

A SaO_2_ of 80%, in long term follow up, has not been shown to negatively impact cognitive function and, therefore should not be considered clinically problematic during V-A ECMO, provided that both ECMO flow and hemoglobin concentration remain adequate [25]. In DH, the upper body extracts oxygen from a systemic arterial saturation of approximately 80%, whereas the lower body, perfused by ECMO, begins oxygen extraction from an arterial saturation of nearly 100%. Consequently, superior vena cava oxygen saturation (SsvcO_2_) will be lower than SivcO_2_, reflecting differential oxygen extraction. As long as an adequate volume of oxygenated blood continues to enter the right heart, leading to an incremental rise in SaO_2_, regional oxygen delivery and consumption reach a new steady state equilibrium. In FDH, upper body SaO_2_ can decline to critically low levels (30–50%) and is confirmed when SaO_2_ measured in the upper body is lower than pre-oxygenator arterial saturation (SpreO_2_). The development of FDH is contingent upon three key factors: (1) effective venous drainage from inferior vena cava (IVC), (2) minimal or absent pulmonary oxygen transfer and (3) a distal displacement of the mixing zone (M-zone) within the aorta, thereby restricting oxygenated blood delivery to the upper body. The primary determinant of this phenomenon is an imbalance between oxygen consumption and total oxygen delivery (DO_2_ ECMO+ DO_2_ cardiac output) to the upper circulation [4]. The anatomical position of the M-zone is highly dynamic and influenced by ECMO flow (Q ECMO), native cardiac output (CO) and both systemic and organ specific vascular resistance [26]. The critical threshold for FDH often arises when ECMO derived oxygenated blood fails to reach the aortic arch, particularly the left subclavian artery. As a result, perfusion of the descending aorta become insufficient, leading to inadequate oxygen transport to the superior vena cava, further exacerbating hypoxia in the upper body.

### 2.3 M-zone

**Fig. 3.**
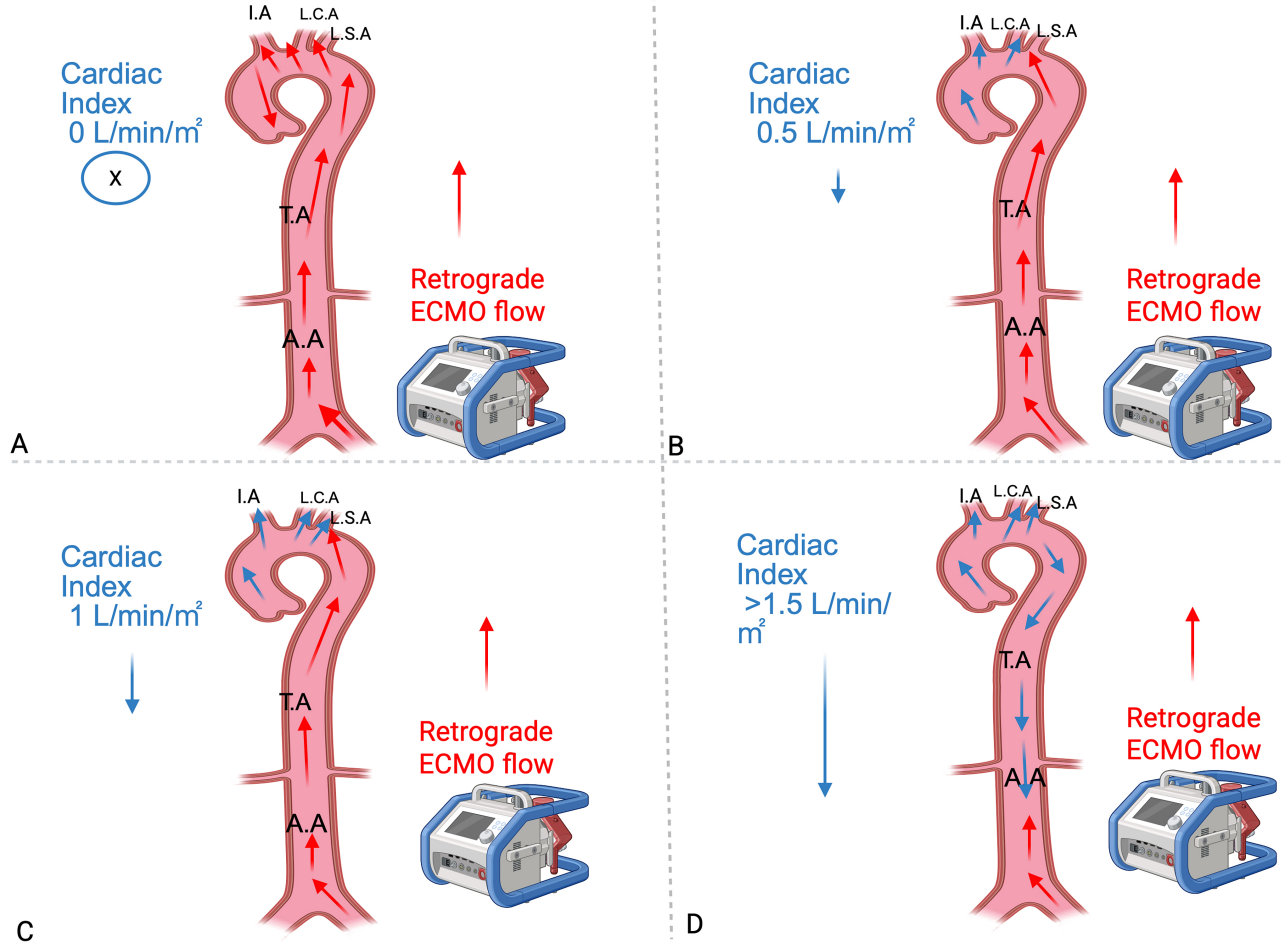
**Differential aortic perfusion zones based on residual cardiac function during F-F V-A ECMO support and mixing zone**. The mixing site location varies with residual cardiac function: stronger native output shifts it distally, while cardiac failure moves it proximally. Blue arrows (native flow) and red arrows (ECMO flow) visualize hemodynamic competition. The position of the M-zone determines hypoxemia risk. (A) At 0 L/min*m^2^ C.I, retrograde ECMO flow perfuses the entire aorta (abdominal, thoracic) and supra-aortic branches (L.S.A, L.C.A, I.A). (B) At 0.5 L/min*m^2^ C.I, retrograde ECMO flow supplies the abdominal/thoracic aorta and LSA, while native cardiac output perfuses the L.C.A and I.A. (C) At 1 L/min*m^2^ C.I, retrograde ECMO flow perfuses the abdominal/thoracic aorta and part of L.S.A, with native flow perfusing part of L.S.A, L.C.A and I.A. (D) At >1.5 L/min*m^2^ C.I, native cardiac output supplies most territories (I.A, L.C.A, L.S.A, thoracic aorta and part of abdominal aorta) while retrograde ECMO flow is restricted to part of abdominal aorta. F-F V-A ECMO, femoro-femoral veno-arterial extracorporeal membrane oxygenation; C.I, cardiac index; I.A, innominate artery; L.C.A, left carotid artery; L.S.A, left subclavian artery; T.A, thoracic aorta; A.A, abdominal aorta; M-zone, mixing-zone. X indicates the absence of native cardiac flow. This figure was created with BioRender (http://www.biorender.com/).

In peripheral V-A ECMO, retrograde arterial inflow establishes a hemodynamic mixing zone (M-zone) within the aorta, representing an equilibrium between antegrade CO and retrograde ECMO flow, modulated by vascular resistance. The precise location of M-zone is dictated by the relative flow velocities, volumes and the anatomical dimension of the vascular segments involved. Notably, evidence suggests that the M-zone shifts distally along the aorta with increasing myocardial contractility and decreasing ECMO support [27] (Fig. 3).

The clinical significance of M-zone positioning presents a complex management challenge. In patients receiving full ECMO support, studies indicate that coronary perfusion may be suboptimal, implying that some degree of aortic valve opening is desirable to ensure myocardial oxygenation [28]. However, in cases of severe pulmonary dysfunction, the oxygen saturation of LV output is markedly reduced. Consequently, even if antegrade coronary perfusion is maintained, myocardial oxygen delivery remains insufficient unless the M-zone is positioned proximally enough to enhance coronary arterial blood oxygenation. Conversely, if the M-zone is displaced too distally, the risk of cerebral, upper body and potentially even renal hypoxia increases, contingent upon its relative position. The dynamic nature of this phenomenon underscores the complexity of M-zone management, as its location is intrinsically linked to LV function, which fluctuates in response to the patient’s evolving clinical status [29]. Prisco *et al*. [30], developed a three-dimensional mathematical model of aorta and its major branching vessels based on human cadaveric anatomical data to simulate physiologically relevant blood flow dynamics in patients supported by peripheral V-A ECMO. Their analysis explored the interplay between residual cardiac function and ECMO derived flow across major aortic branch vessels, particularly the cerebral and coronary circulations. With a residual cardiac index (CI) of 0 L/min*m^2^ all blood flow at each of the three outlets (innominate, left carotid, and left subclavian artery) originated from the VA-ECMO circuit. When C.I increased to 0.5 L/min*m^2^ (indicative of severe cardiogenic shock), ECMO flow predominantly perfused the left subclavian artery, while cerebral circulation (innominate and left carotid artery) was mainly supported by native cardiac output. As CI continued to increase, a progressive displacement of ECMO derived flow was observed, culminating in complete exclusion of ECMO blood from the cerebral circulation at a cardiac index of 1.5 L/min*m^2^. The coronary arteries, being anatomically closest to the aortic annulus, exclusively received native cardiac output as soon as even minimal residual function was present (>0.5 L/min*m^2^). Notably the degree to which residual cardiac output displaced ECMO flow correlated with the anatomical distance of each vessel from the aortic root, with the left subclavian requiring twice the residual cardiac output to eliminate ECMO flow compared to the innominate artery. These findings highlight that, despite the brain’s vulnerability to hypoxia, myocardial perfusion is more immediately impacted in the setting of DH. Furthermore, the study demonstrated that increasing ECMO flow shifted the mixing zone more proximally along the aorta. At lower ECMO flow rates, mixing occurred in the abdominal aorta, whereas increasing ECMO support progressively displaced the M-zone into the thoracic aortic arch (when C.I is held constant 1 L/min*m^2^). These findings highlight the dynamic nature of oxygen delivery in V-A ECMO and the necessity of individualized flow titration to optimize end organ perfusion. Notably, excessive retrograde ECMO flow should be avoided, as it may impose an increased afterload on the left ventricle, thereby hindering myocardial recovery.

Presently, no standardized clinical modality exists for real time localization of the mixing zone in V-A ECMO patients. While contrast-enhanced computed tomography (CT) or angiography has been empirically employed for this purpose, these radiographic techniques carry significant limitations including exposure to ionizing radiations and nephrotoxic risks associated with iodinated contrast administration [31, 32, 33]. Buchtele *et al*. [34] investigated the feasibility and safety of contrast enhanced ultrasound (CEUS) in identifying aortic mixing zone in patients on peripheral V-A ECMO, using SonoVue contrast and performing transesophageal echocardiography and transabdominal sonography. Their study demonstrates that CEUS effectively visualizes the interface between native cardiac output and retrograde ECMO flow, providing a non invasive tool for assessing dynamic flow patterns. The results suggest that CEUS could aid in optimizing ECMO management by guiding flow adjustments to prevent DH while maintaining a favorable safety profile. Similarly, a study conducted by Reddan *et al*. [35] demonstrates that ultrasonographic assessment of aortic flow can be useful for identifying the mixing zone. In this context, Giustiniano and Cecconi [36] presented a case report suggesting the potential utility of suprasternal transthoracic echocardiography for localizing the M-zone at the level of the aortic arch. The authors describe a characteristic ultrasonographic finding, a proximal concave plume, hypothesized to represent a “smoke effect” generated by the interface between anterograde native and retrograde ECMO flows. This suprasternal approach, although currently underutilized due to limited validation in clinical studies, may represent a valuable, radiation-free, bedside modality for the non-invasive assessment of the aortic mixing zone.

### 2.4 Differential Hypoxia Management 

**Fig. 4.**
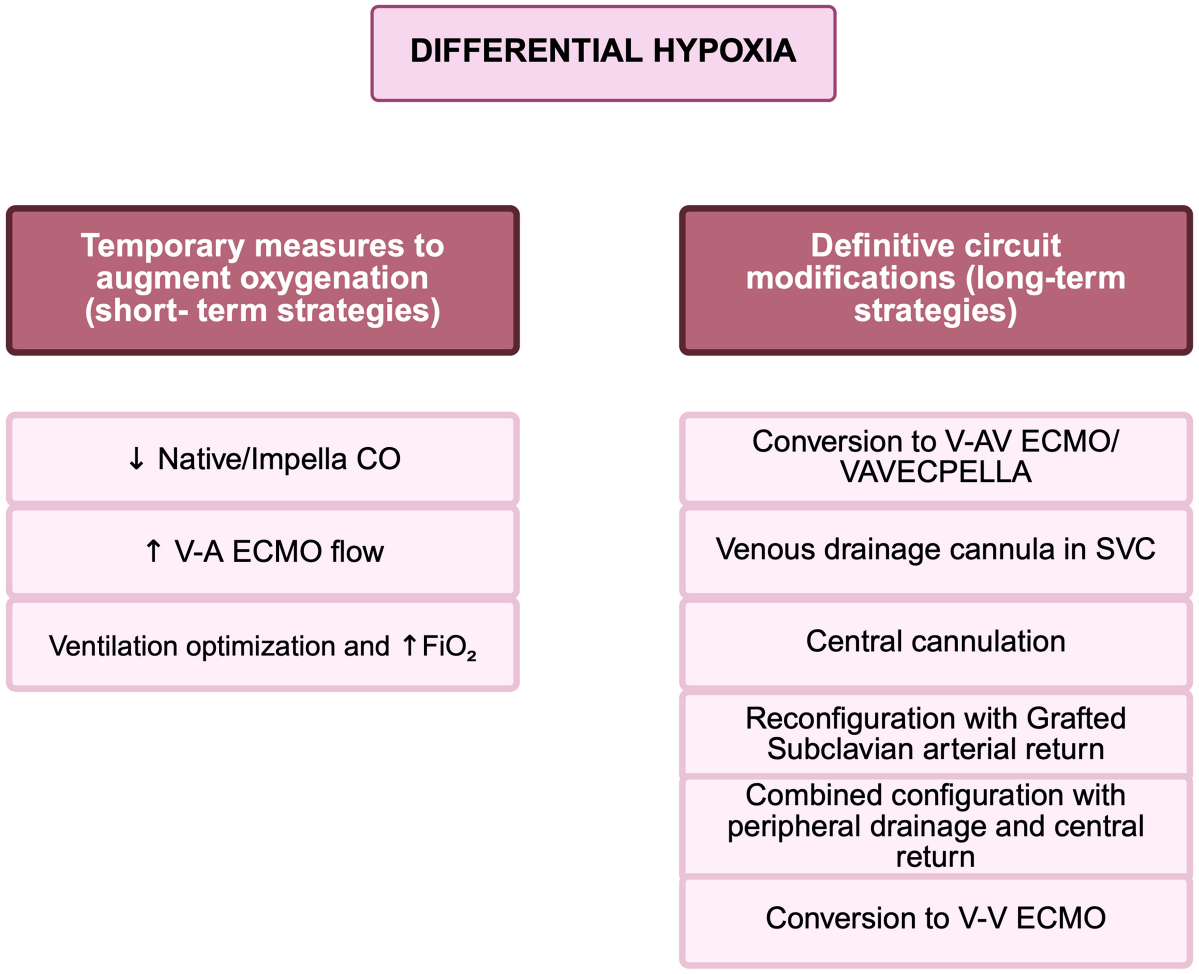
**Management of differential hypoxia during F-F V-A ECMO or ECPELLA support**. Short-term and long-term strategies. Short term strategies aim to restore adequate upper body oxygenation by minimizing the competition between native cardiac output/Impella flow and retrograde ECMO flow. These include reduction of native cardiac output or Impella flow, augmentation of ECMO flow (>4 L/min), and optimization of oxygenation through ventilator settings and FiO_2_. Long term strategies are indicated when DH persists despite initial measures. These include circuit reconfiguration, such as conversion to V-AV ECMO or VAVECPELLA setup, revision of venous drainage cannulation (e.g., adding central or SVC drainage), or transition to V-V ECMO in cases of adequate myocardial recovery. Management decisions should be guided by continuous monitoring of cerebral oximetry (rSO_2_), lactate clearance, and echocardiographic indicators of cardiac function and mixing zone position. F-F V-A ECMO, femoro-femoral veno-arterial extracorporeal membrane oxygenation; ECPELLA, ECMO with Impella support; V-V ECMO, veno-venous ECMO; V-AV ECMO, veno-arterial-venous ECMO; VAVECPELLA, veno-arterial-venous ECMO with Impella; CO, cardiac output; SVC, superior vena cava; SaO_2_, oxygen saturation of arterial blood; rSO_2_, regional oxygen saturation; FiO_2_, fraction of inspired oxygen; ↑, increase; ↓, decrease. This figure was created with BioRender (http://www.biorender.com/).

Prompt recognition of differential hypoxia is critical to guide therapeutic intervention and to optimize clinical management in V-A ECMO patients (Table 1, Fig. 4). Clinical decision-making should consider the anatomical location of the hemodynamic mixing-zone (M-zone) and the severity of pulmonary dysfunction.

**Table 1.** **Diagnostic tools, therapeutic strategies and clinical indication for DH during V-A ECMO or ECPELLA support**.

Diagnostic tools		Strategy	Purpose
Cerebral oximetry (NIRS)		Regional oxygen saturation rSO_2_ monitoring	Early detection of cerebral desaturation (rSO_2_ <50%)
Arterial blood gas (ABG)		SaO_2_ and pO_2_ comparison at multiple sites (right radial, left radial, femoral)	Identifies oxygenation gradients and confirms DH diagnosis
Pulse oximetry (spO_2_)		Differential spO_2_ between right and left upper limbs/lower limbs	Non-invasive screening for asymmetrical oxygenation
Echocardiography		Identification of mixing zone and native heart function	Locates mixing zone; evaluates ejection and ventricular recovery
Lactate levels		Serial lactate measurement	Marker of systemic and regional hypoperfusion
Short-term strategies			
	Increase ECMO flow	Target >4.5–5 L/min when possible	Enhances retrograde perfusion to shift mixing zone proximally
	Optimize oxygenation	FiO_2_ 100%, PEEP optimization, recruitment maneuvers	Improves pulmonary oxygen exchange
	Reduce native cardiac output	Reduce inotropes, beta-blockers administration	Minimizes competition with ECMO flow
	Adjust Impella flow (in ECPELLA)	Titrate Impella support to balance LV unloading and cerebral oxygenation	Prevents dominance of deoxygenated output
Long-term strategies			
	Circuit reconfiguration	V-AV ECMO, VAVECPELLA configuration	Ensures oxygenated blood reaches upper body (including brain and coronaries)
	Cannulation revision	Central aortic return or SVC drainage	Improves antegrade flow distribution or drainage efficiency
	Conversion to V-V ECMO	After myocardial recovery	Eliminates differential flow conflict and improves oxygenation
Clinical monitoring			
	Neurologic status	Agitation, confusion, seizures	Suggest cerebral hypoxia
	Electrocardiographic changes	ST elevation, arrhythmias	Indicates myocardial ischemia
	Hemodynamic instability	Inadequate MAP despite flows	May signal coronary malperfusion

Overview of key diagnostic tools, short- and long-term therapeutic approaches, and clinical signs associated with DH. The content aims to support decision-making when competing blood flows compromise oxygen delivery to the brain and myocardium. NIRS, near-infrared spectroscopy; ABG, arterial blood gas; V-A ECMO, veno-arterial extracorporeal membrane oxygenation; ECPELLA, ECMO with Impella support; V-V ECMO, veno-venous ECMO; V-AV ECMO, veno-arterial-venous ECMO; VAVECPELLA, veno-arterial-venous ECMO with Impella; SaO_2_, oxygen saturation of arterial blood; pO_2_, arterial oxygen partial pressure; spO_2_, peripheral oxygen saturation; rSO_2_, regional oxygen saturation; SVC, superior vena cava; LV, left ventricle; PEEP, positive end-expiratory pressure; FiO_2_, fraction of inspired oxygen; ST, ST segment of the electrocardiogram; MAP, mean arterial pressure.

When managing hypoxia related complications in these patients, clinicians must consider the predicted anatomical location of the hemodynamic mixing zone.

#### 2.4.1 Temporary Measure to Augment Oxygenation (Short Term Strategies)

While preparing for more definitive interventions, such as ECMO circuit reconfiguration, several temporizing measures can be implemented [4]:

• Increased V-A ECMO pump flow: augmenting the pump flow to enhance retrograde oxygenated blood delivery.

• Negative inotropic agents: administered to reduce native cardiac output, thereby limiting antegrade ejection of desaturated blood from the left ventricle.

• Ventilatory optimization: increasing fraction of inspired oxygen (FiO_2_) to counteract life-threatening hypoxemia. This must be used with caution due to the risk of ventilator induced lung injury (VILI) caused by oxygen toxicity, volotrauma, absorption atelectasis from nitrogen wash out [37].

These are solely temporarizing strategies, as prolonged use may ultimately impair myocardial recovery.

#### 2.4.2 Long Term Circuit Modifications 

• Conversion from V-A to V-AV ECMO: placement of an additional return cannula in the superior vena cava (SVC), converting V-A ECMO to Veno-Arterial-Venous (V-AV) configuration [38, 39, 40, 41]. This approach involves transitioning from V-A to V-AV ECMO by introducing a secondary return cannula into the SVC via the jugular vein, thereby enhancing cerebral oxygenation by redistributing extracorporeal flow between the upper and the lower body. This modification reduces oxygen delivery (DO_2_ ECMO) to the lower body. However, V-AV ECMO presents several challenges, including recirculation which is determined by veno-venous (VV) component and can diminish DO_2_ ECMO. Attempts to counteract this by increasing flow may further exacerbate both recirculation and hemolysis. Additionally, an unpredictable flow partitioning may occur, where the balance between VV and VA support is dynamic and is influenced by factors such as arterial pressure gradient, intrathoracic and venous pressures, regional vascular resistance and cannula design. To maintain equilibrium, the venous return tubing should have higher resistance than arterial circuit, however manual adjustments (e.g., gate clamps) increase the risk of thrombosis. Flow regulation via a roller pump may mitigate this risk. Finally, Y connectors and additional tubing predispose to platelet activation, visible clot formation and red blood cell damage, thereby increasing the risks of coagulation and hemolysis.

In a study by Zhao *et al*. [42] involving seven adult sheep with induced lung failure, the hybrid ECMO circuit was evaluated to decrease amount of recirculation (a complication of V-AV ECMO). This setup combines V-A ECMO with a VV component, utilizing the Avalon Elite double lumen cannula (DLC) positioned from the IVC to the SVC through the RA and a 17F infusion cannula in the femoral artery. Total ECMO flow was maintained between 2.8 to 3.3 L/min, with the VV component adjusted incrementally from 0% to 50% of the total flow. Findings demonstrated that introducing VV flow significantly elevated LV blood oxygen saturation (from 70% ± 8 at 0% VV flow to 96% ± 6 at 50% VV flow). Notably, even a modest VV flow of 10% led to a statistically significant improvement in LV oxygenation. The study concluded that Avalon Elite DLC—based hybrid ECMO circuit effectively mitigates differential hypoxia by allowing flexible distribution of oxygenated blood between VA and VV pathways, ensuring adequate oxygen delivery to vital organs, particularly the heart and the brain.

• Modified venous drainage strategy (SVC/right atrium (RA) drainage):

Hou *et al*. [43] studied the effects of SVC drainage on upper body oxygenation in a sheep model of V-A ECMO. They found that SVC drainage improved cerebral and myocardial oxygenation compared to traditional IVC drainage, reducing DH. Similarly, Falk *et al*. [44] found that repositioning the V-A ECMO drainage cannula from the IVC to the SVC significantly improved upper body oxygenation in patients with severe DH [39].

Draining low saturated blood from the SVC/RA enhances ECMO efficiency by simplifying the circuit and reducing the risk of unpredictable flow distribution and coagulation complications seen with V-AV configuration. This strategy minimizes the risk of FDH, as blood flow is quickly established and “physiologic” DH is less pronounced, often eliminating the need for an additional return cannula. The drainage from the SVC/RA allows oxygen rich blood from the IVC to enter RA, sustaining cardiac output. This improves both pulmonary arterial oxygen saturation (SPaO_2_) and systemic arterial SaO_2_. In cases of decreased cardiac output, retrograde ECMO flow reaches the upper regions, but SsvcO_2_ remains similar or lower than SivcO_2_.

• Grafted Subclavian arterial return [45]: cannulation via a vascular graft anastomosed to the subclavian artery eliminates the risk of FDH but may cause DH due to hyper-oxygenation of the right arm. To prevent this, a snare is often used around the subclavian artery, though this increases the risk of clot formation and thromboembolism. Additionally subclavian artery cannulation carries the risk of cerebral emboli, as ECMO perfuses the aortic arch.

• Central cannulation [46, 47]: requires sternotomy, with cannulae placed in the atria, ventricles and major vessels including the aorta. While larger diameter cannula allows for high flows even with low venous filling, this configuration increases the risks of bleeding and cerebral embolism, both thrombotic and gaseous.

• Hybrid configuration (peripheral drainage and central return) [48]: combines peripheral jugular drainage from SVC/RA with central return via a chimney graft to the innominate artery for reinfusion. Often used as bridge to transplantation, this setup carries similar cerebral risks as central cannulation.

• Conversion to Veno-venopulmonary (V-VP) ECMO: A reconfiguration of V-A ECMO into V-VP ECMO circuit may be considered when both right ventricular and respiratory support are required [49, 50].

• Arterial cannula tip repositioning [51]: the study by Wickramarachchi *et al*. [51] explores how arterial cannula tip positioning affects upper body oxygenation during V-A ECMO. Using computational simulations, they analyzed four positions (iliac artery, abdominal aorta, descending aorta and aortic arch) under different ECMO support levels. Result shows that only aortic arch placement ensures consistent oxygen delivery to the brain, while lower position require maximal ECMO support to achieve similar perfusion. This study highlights the importance of optimal cannula positioning to prevent DH and improve cerebral oxygenation.

• Conversion to V-V ECMO: when circulatory support is no longer required. Secondary right heart failure remains a potential risk.

### 2.5 Differential Hypoxia in ECPELLA

The combined use of V-A ECMO and Impella (ECPELLA or ECMELLA configuration) has emerged as a pivotal strategy for managing refractory cardiogenic shock. This dual mechanism approach provides synergistic benefits because V-A ECMO maintains systemic perfusion while Impella actively unloads the left ventricle, offering superior hemodynamic decompression and unloading compared to other surgical venting techniques (e.g., pulmonary artery or LV apical venting) [52, 53] (Fig. 1B).

However, in patients with concomitant severe pulmonary dysfunction, ECPELLA presents a unique physiological challenge. Similar to the native heart, the Impella device withdraws hypoxemic blood from LV and reinfuses it into the ascending aorta, generating competing oxygen gradients that may exacerbate DH. This phenomenon preferentially compromises coronary and cerebral oxygenation due to the anatomical proximity of coronary ostia to Impella outflow tract and insufficient mixing between oxygenated (ECMO derived) and deoxygenated (Impella derived) blood streams (Fig. 1B). The observational study by Shibao *et al*. [54] investigates the incidence of DH in cardiac arrest patients treated with V-A ECMO combined with Impella. The finding revealed a significant increase in differential hypoxia 96 hours after ECPELLA initiation, requiring conversion to V-AV ECMO.

Ushijima *et al*. [55] present a case involving a 70-year-old male with cardiogenic shock due to fulminant myocarditis initially managed with ECPELLA. The patient develops significant differential hypoxia with upper body desaturation. To resolve this, the ECMO configuration was transitioned from V-A to V-AV, resulting in a VAVECPELLA setup. This modification effectively resolved the DH, facilitating successful weaning from mechanical circulatory support. VAVECPELLA may enhance myocardial recovery by mitigating coronary desaturation associated with ECPELLA support.

Giunta *et al*. [56] present two cases of cardiogenic shock managed with ECPELLA. Both patients developed DH. The author emphasizes the importance of monitoring arterial blood gases from multiple sites (right and left radial line and ECMO arterial line) to identify the M-zone location. Adjusting the flows of V-A ECMO and Impella can reposition the M-zone, optimizing delivery to vital organs but sometimes this is not enough, necessitating the ECPELLA reconfiguration.

Neidlin *et al*. [57] conducted a computational study to analyze aortic hemodynamics and oxygenation during V-A ECMO with and without Impella support. Using a human aorta model, they evaluated various cannula tip positions (iliac artery, abdominal aorta, thoracic aorta and descending aorta) and ECMO support levels (50%, 75% and 90%) with a total blood flow of 6 L/min. The study found that more proximal cannula placements (closer to the heart) improved oxygenation of the coronary and supra-aortic vessels, especially under lower ECMO support levels (50% and 75%). Additionally, incorporating Impella support reduced afterload by 8–17 mmHg, but also decreased oxygenation to coronary and supra-aortic vessels to levels similar to 50% V-A ECMO support.

In summary, the Impella, like the native heart, can compete with the retrograde aortic flow mediated by V-A ECMO resulting in DH when associated with severe pulmonary compromise. Under the ECPELLA setup, ECMO V-A ensures management of severe cardiogenic shock by providing adequate peripheral perfusion, while the Impella reduces afterload and ventricular filling pressures and prevents ventricular over distension (through its trans-aortic mechanism of withdrawing blood from the left ventricle and ejecting it into the ascending aorta). However, the ejection of poorly oxygenated blood into the ascending aorta (as the lungs fail to oxygenate the blood, effectively functioning as a passive conduit) leads to differential hypoxia. In this condition, the upper body becomes desaturated due to the anterograde flow mediated by Impella, while the lower body remains well oxygenated by V-A ECMO. Therefore, recognizing this condition is crucial to prevent complications (neurological and myocardial damage) and ensure early diagnosis, which can be achieved through bilateral pulse oximetry, blood gas sampling from the right and left radial artery lines and from ECMO arterial line, the use of NIRS and, possibly echocardiography to identify the M-zone. Treatment should be tailored to the patient, with therapeutic strategies ranging from flow titration of V-A ECMO and Impella to reconfiguration of ECPELLA to VAVECPELLA.

## 3. Conclusions

Differential hypoxia in V-A ECMO and ECPELLA configurations remains a critical and under-recognized challenge in advanced cardiopulmonary support. This review underscores the need for continuous, multimodal monitoring and tailored management strategies, as no one size fits all approach exists. The introduction of ECPELLA, while enhancing ventricular unloading, further complicates oxygenation dynamics and requires vigilant assessment of cerebral and myocardial perfusion. Actionable strategies include integrating right radial pressure monitoring, cerebral oximetry, multi-site arterial blood gas analysis, and echocardiographic or contrast-enhanced ultrasound assessment of the mixing zone. In refractory cases, circuit reconfiguration may be necessary, though these solutions carry procedural complexity risk. Major knowledge gaps persist regarding optimal thresholds for intervention, validation of non-invasive imaging tools, and standardized monitoring protocols. Future research should focus on prospective evaluation of these modalities and development of individualized algorithms to guide DH diagnosis and treatment.

Successful management of differential hypoxia demands more than technological precision; it requires dynamic clinical reasoning and interdisciplinary collaboration to navigate an evolving physiological landscapes. DH serves as a powerful reminder that in cardiac critical care, solving one problem often uncovers another. Clinical expertise relies not only on the deployment of advanced technologies but equally on a deep understanding of their complex interplay with the fragile physiology of critically ill patients.

## Abbreviations

V-A ECMO, veno-arterial extracorporeal membrane oxygenation; DH, differential hypoxia; IVC, inferior vena cava; SVC, superior vena cava; RA, right atrium; LV, left ventricle; V-AV ECMO, veno-arterial venous membrane oxygenation; ECPELLA, ECMO with Impella support; DLC, dual lumen cannula; FDH, fulminant differential hypoxia; CO, cardiac output; CI, cardiac index; SaO_2_, systemic arterial saturation; SPaO_2_, pulmonary arterial saturation; SsvcO_2_, superior vena cava oxygen saturation; SivcO_2_, inferior vena cava saturation; DO_2_ ECMO, oxygen delivery via ECMO.
